# Crystal structure of 4-({(1*E*,2*E*)-3-[3-(4-fluoro­phen­yl)-1-isopropyl-1*H*-indol-2-yl]allyl­idene}amino)-1*H*-1,2,4-triazole-5(4*H*)-thione

**DOI:** 10.1107/S205698901502201X

**Published:** 2015-11-25

**Authors:** Ajaykumar D. Kulkarni, Md. Lutfor Rahman, Mashitah Mohd. Yusoff, Huey Chong Kwong, Ching Kheng Quah

**Affiliations:** a Department of Chemistry, KLS’s Gogte Institute of Technology, Jnana Ganga, Udyambag, Belagavi 590 008 Karnataka, India; b University Malaysia Pahang, Faculty of Industrial Sciences and Technology, 26300 Gambang, Kuantan, Pahang, Malaysia; c School of Chemical Sciences, Universiti Sains Malaysia, 11800 USM, Penang, Malaysia; d X-ray Crystallography Unit, School of Physics, Universiti Sains Malaysia, 11800 USM, Penang, Malaysia

**Keywords:** crystal structure, 1,2,4-triazole-3-thione, indole, Schiff base, N—H⋯N hydrogen bonds, C—H⋯π inter­actions, π–π inter­actions

## Abstract

The asymmetric unit of the titled compound, C_22_H_20_FN_5_S, comprises two independent mol­ecules (*A* and *B*), both of which have a *trans* conformation with respect to the methene C=C [1.342 (2) and 1.335 (2) Å] and the acyclic N=C [1.283 (2) and 1.281 (2) Å] bonds. In mol­ecule *A*, the triazole ring makes dihedral angles of 55.01 (12) and 18.17 (9)° with the benzene and indole rings, respectively. The corresponding dihedral angles for mol­ecule *B* are 54.54 (11) and 14.60 (10)°, respectively. In the crystal, mol­ecules are consolidated into –*A*–*B*–*A*–*B*– chains along [010] *via* N—H⋯N hydrogen bonds. The chains are further linked into layers parallel to the *ac* plane *via* π–π inter­actions involving inversion-related triazole rings [centroid–centroid distances = 3.3436 (11)–3.4792 (13) Å].

## Abstract 2

The asymmetric unit of the title compound comprises two independent mol­ecules which exist in the *trans* conformation with respect to the methene C= C and the acyclic N=C bonds. In the crystal, mol­ecules are linked *via* N—H⋯N hydrogen bonds, forming chains along the *b*-axis direction.

## Chemical context   

The chemistry of 1,2,4-triazole derivatives has attracted widespread attention due to their diverse biological activities and because they are a new class of anti­microbial agents (Sun *et al.*, 2004 ▸; Verreck *et al.*, 2003 ▸); for example fluconazole and itraconazole are used as anti­microbial drugs. Hence, metal complexes of Schiff bases derived from 1,2,4-triazole derivatives have been the subject of considerable study (Ozarowski *et al.*, 1991 ▸; Cornelissen *et al.*, 1992 ▸; Varma *et al.*, 1992 ▸; Mishra & Said, 1996 ▸). A number of metal complexes with 1,2,4-tri­azole Schiff bases have been reported from our laboratory (Yadawe & Patil, 1997 ▸; Avaji *et al.*, 2006 ▸; Kulkarni *et al.*, 2009 ▸, 2011 ▸). In addition to this isatin, which is an endogenous indole, and its derivatives have been shown to exhibit a wide range of biological activities (Daisley & Shah, 1984 ▸; Pandeya *et al.*, 1999*a* ▸,*b* ▸; Cerchiaro & Ferreira, 2006 ▸; Sridhar *et al.*, 2002 ▸).Since triazoles are heterocyclic compounds and Schiff bases derived from isatin often act as versatile chelating agents and exhibit promising bioactivities, it is likely that a Schiff base derived from fluvastatin–triazole might also exhibit useful biological activities. In this way, it was planned to prepare a Schiff base which possesses both nitro­gen and sulfur coordination cites so that it might coordinate effectively to metal ions.

## Structural commentary   

**Figure 1:**
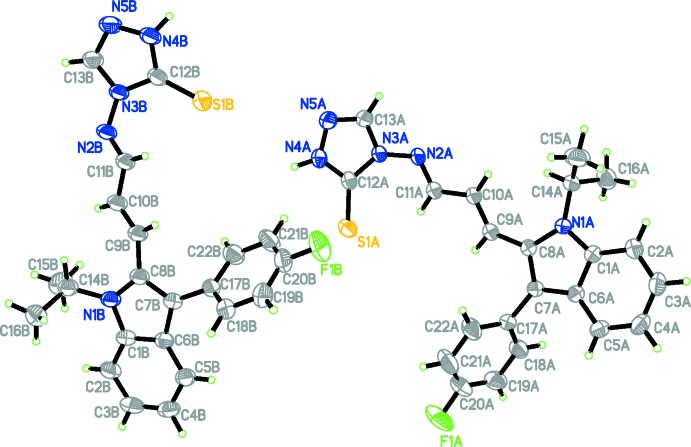
The mol­ecular structure of the two independent mol­ecules (*A* and *B*) of the title compound, showing the atom labelling. Displacement ellipsoids are drawn at the 30% probability level.

The asymmetric unit of the title compound (Fig. 1 ▸) is comprised of two independent mol­ecules (*A* and *B*). Both mol­ecules have a *trans* conformation with respect to the methene C=C [1.342 (2) and 1.335 (2) Å in mol­ecules *A* and *B*, respectively] and the acyclic N=C bonds [1.283 (2) and 1.281 (2) Å in mol­ecules *A* and *B*, respectively]. The indole rings are almost planar [maximum deviations of 0.017 (2) Å for atom C8*A* in mol­ecule *A* and 0.027 (2) Å for atom N1*B* in mol­ecule *B*]. In mol­ecule *A*, the triazole ring makes dihedral angles of 55.01 (12) and 18.17 (9)°, respectively, with the fluoro­phenyl and indole rings [54.54 (11) and 14.60 (10)°, respectively, in mol­ecule *B*]. The indole and fluoro­phenyl rings are inclined to one another by a dihedral angle of 64.78 (9)° [55.21 (8)° in mol­ecule *B*]. The bond lengths and angles in the triazole-thione moiety of the title compound are comparable to those reported for related compounds (Fun *et al.*, 2008 ▸; Kulkarni *et al.*, 2015 ▸).
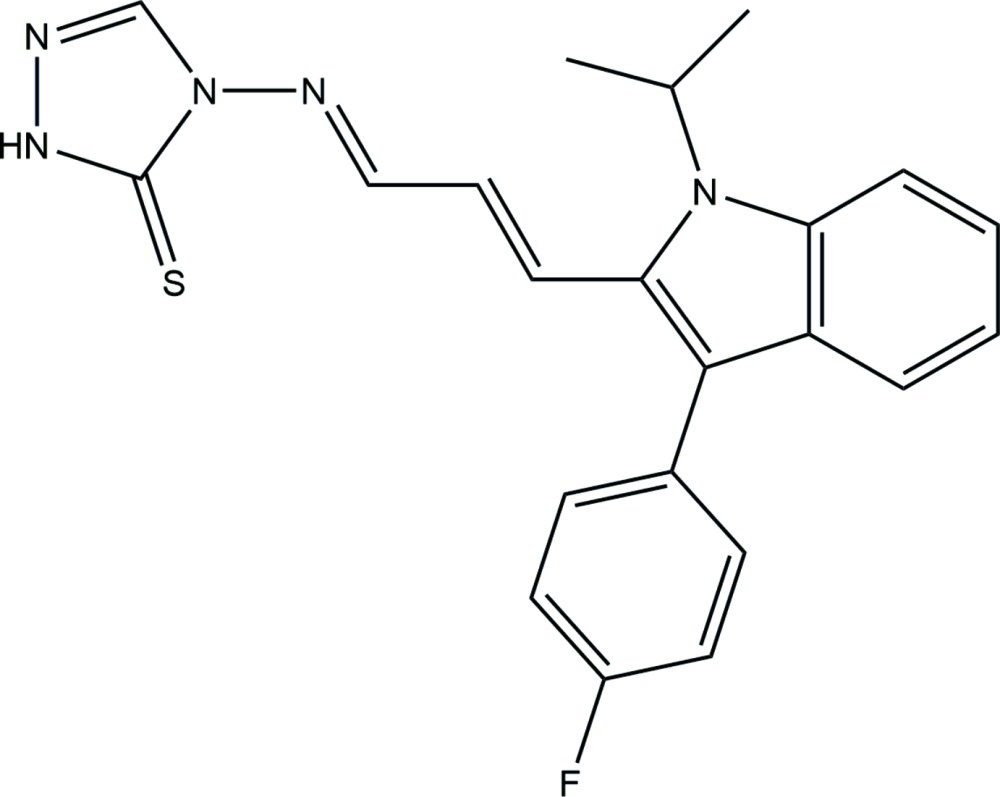


## Supra­molecular features   

**Figure 2:**
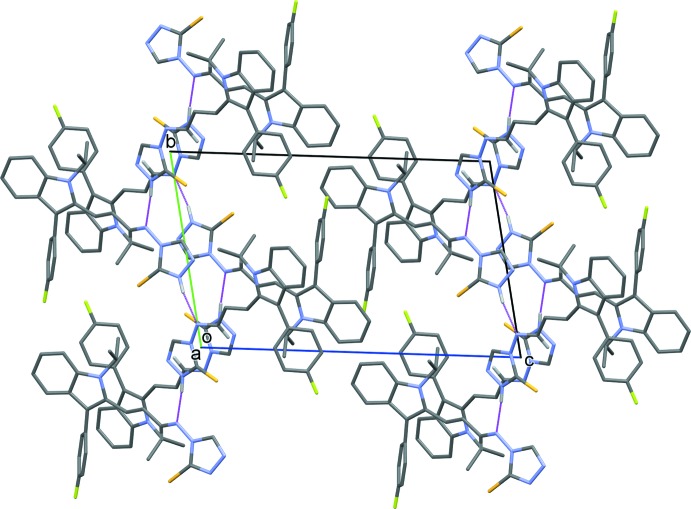
The crystal packing of the title compound viewed along the *a* axis. The N—H⋯N hydrogen bonds are shown as dashed lines (see Table 1 ▸). H atoms not involved in hydrogen bonding have been omitted for clarity.

In the crystal, mol­ecules *A* and *B* are consolidated into –*A*–*B*–*A*–*B*– chains along [010] *via* N—H⋯N hydrogen bonds (Table 1 ▸ and Fig. 2 ▸). The chains are linked *via* C—H⋯π inter­actions (Table 1 ▸) and slipped parallel π–π inter­actions, involving inversion-related triazole rings, forming layers parallel to the *ac* plane [*Cg*4⋯*Cg*4^i^ = 3.3436 (11) Å; *Cg*4 is the centroid of ring N3*A*–N5*A*/C12*A*/C13*A*; inter­planar distance = 3.2317 (8) Å; slippage = 0.858 Å; symmetry code: (i) −*x*, −*y* + 1, −*z*, and *Cg*5⋯*Cg*5^ii^ = 3.4792 (13) Å; *Cg*5 is the centroid of ring N3*B*–N5*B*/C12*B*/C13*B*; inter­planar distance = 3.4194 (9) Å; slippage = 0.642 Å; symmetry code: (ii) −*x* + 2, −*y* + 2, −*z*].

## Database survey   

A search of the Cambridge Structural Database (Version35.6, last update May 2015; Groom and Allen, 2014 ▸) using 4-(λ^1^-azan­yl)-5-methyl-2,4-di­hydro-3*H*-1,2,4-triazole-3-thione as the main skeleton, revealed the presence of 57 structures containing the triazole-thione moiety but only four structures containing the fluvastatin nucleus. These include 5-(3-(4-fluoro­phen­yl)-1-isopropyl-1*H*-indol-2-yl)-1-(*X*)penta-2,4-diene-1-one (Kalalbandi *et al.*, 2015 ▸), where *X* = 4-nitro­phenyl (NUHNAH), 2-hy­droxy­phenyl (NUHNEL), 4-meth­oxy­phenyl (NUHNIP) and 4-chloro­phenyl (NUHNOV). In the four compounds the 4-fluoro­phenyl ring of the fluvastatin nucleus is inclined to the indole ring by dihedral angles ranging from *ca* 46.66 to 68.59°, compared to 55.01 (12) and 55.21 (8)° for the title compound.

## Synthesis and crystallization   

The title compound was synthesized by refluxing a hot ethano­lic solution (30 ml) of 3-substituted-4-amino-5-mercapto-1,2,4-triazole (0.01 mol) and a hot ethano­lic solution (30 ml) of fluvastatin (0.01 mol) for 4–5 h with addition of a catalytic amount of concentrated hydro­chloric acid. The product obtained after evaporation of the solvent was filtered and recrystallized from hot ethanol. Single crystals were obtained by slow evaporation of a solution in chloro­form (yield 74%; m.p. 464 K). ^1^H NMR (D6-DMSO): 10.4 (*s*, 1H, NH), 10.01 (*s*, 1H, CH=N), 7.1–7.7 (*m*, 8H, Ar–H), 7.3 (*s*, 1H, triazole-H), 6.47–6.55 (*d*, 2H, –CH=CH–), 6.47–6.56 (*s*, 6H, isopropyl group). IR (KBr, cm^−1^): 3224, 3176 (N—H), 2754 (C—H), 1616 (C=N), 1600–1500 (C=C), 1105 (C=S). FAB–MS: *m*/*z* 405. Analysis: observed(calculated) C, 65.11 (65.18); H, 4.81 (4.93); N, 17.19 (17.28).

## Refinement   

Crystal data, data collection and structure refinement details are summarized in Table 2 ▸. C-bound H atoms were positioned geometrically [C—H = 0.95–0.97 Å] and refined using a riding model with *U*_iso_(H) = 1.2 or 1.5*U*_eq_(C). All N-bound H atoms were located from a difference Fourier map and freely refined [N—H = 0.90 (3)–0.91 (3) Å].

## Supplementary Material

Crystal structure: contains datablock(s) I. DOI: 10.1107/S205698901502201X/su5237sup1.cif

Structure factors: contains datablock(s) I. DOI: 10.1107/S205698901502201X/su5237Isup2.hkl

Supporting information file. DOI: 10.1107/S205698901502201X/su5237Isup3.cml

CCDC reference: 1437565

Additional supporting information:  crystallographic information; 3D view; checkCIF report

## Figures and Tables

**Table 1:** Hydrogen-bond geometry (Å, °) *Cg*1 is the centroid of ring N3*A*–N5*A*/C12*A*/C13*A*, *Cg*2 is the centroid of ring C17*B*–C22*B*, and *Cg*3 is the centroid of ring C1*A*–C6*A*.

*D*—H⋯*A*	*D*—H	H⋯*A*	*D*⋯*A*	*D*—H⋯*A*
N4*A*—H4*AB*⋯N2*B* ^i^	0.90 (2)	2.05 (2)	2.944 (2)	170 (2)
N4*B*—H4*BB*⋯N2*A* ^ii^	0.89 (2)	2.02 (2)	2.906 (2)	170 (3)
C15*B*—H15*E*⋯*Cg*1^iii^	0.96	2.91	3.521 (3)	123
C16*A*—H16*B*⋯*Cg*2^iv^	0.96	2.87	3.716 (2)	148
C21*A*—H21*A*⋯*Cg*3^v^	0.93	2.90	3.668 (2)	140

Symmetry codes: (i) 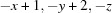; (ii) 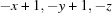; (iii) 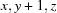; (iv) 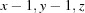; (v) 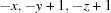.

**Table 2:** Experimental details

Crystal data	
Chemical formula	C_22_H_20_FN_5_S
*M*_r_	405.49
Crystal system, space group	Triclinic, *P* 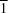
Temperature (K)	297
*a*, *b*, *c* (Å)	9.9283 (4), 11.5343 (5), 18.4694 (7)
α, β, γ (°)	99.8886 (13), 94.9582 (14), 98.4315 (14)
*V* (Å^3^)	2047.54 (14)
*Z*	4
Radiation type	Mo *K*α
μ (mm^−1^)	0.19
Crystal size (mm)	0.66 × 0.60 × 0.46

Data collection	
Diffractometer	Bruker APEXII DUO CCD area detector
Absorption correction	Multi-scan (*SADABS*; Bruker, 2009 ▸)
*T*_min_, *T*_max_	0.730, 0.830
No. of measured, independent and observed [*I* > 2σ(*I*)] reflections	78666, 12002, 7987
*R*_int_	0.048
(sin θ/λ)_max_ (Å^−1^)	0.707

Refinement	
*R*[*F* ^2^ > 2σ(*F* ^2^)], *wR*(*F* ^2^), *S*	0.053, 0.135, 1.02
No. of reflections	12002
No. of parameters	535
H-atom treatment	H atoms treated by a mixture of independent and constrained refinement
Δρ_max_, Δρ_min_ (e Å^−3^)	0.32, −0.35

Computer programs: *APEX2* and *SAINT* (Bruker, 2009 ▸), *SHELXS97* (Sheldrick, 2008 ▸), *SHELXL2013* (Sheldrick, 2015 ▸), *Mercury* (Macrae *et al.*, 2008 ▸) and *PLATON* (Spek, 2009 ▸).

## supplementary crystallographic information

### Crystal data

**Table:**

C_22_H_20_FN_5_S	*Z* = 4
*M**_r_* = 405.49	*F*(000) = 848
Triclinic, *P*1	*D*_x_ = 1.315 Mg m^−^^3^
*a* = 9.9283 (4) Å	Mo *K*α radiation, λ = 0.71073 Å
*b* = 11.5343 (5) Å	Cell parameters from 9367 reflections
*c* = 18.4694 (7) Å	θ = 2.3–27.8°
α = 99.8886 (13)°	µ = 0.19 mm^−^^1^
β = 94.9582 (14)°	*T* = 297 K
γ = 98.4315 (14)°	Block, yellow
*V* = 2047.54 (14) Å^3^	0.66 × 0.60 × 0.46 mm

### Data collection

**Table:**

Bruker APEXII DUO CCD area-detector diffractometer	12002 independent reflections
Radiation source: fine-focus sealed tube	7987 reflections with *I* > 2σ(*I*)
Graphite monochromator	*R*_int_ = 0.048
φ and ω scans	θ_max_ = 30.2°, θ_min_ = 1.8°
Absorption correction: multi-scan (*SADABS*; Bruker, 2009)	*h* = −13→14
*T*_min_ = 0.730, *T*_max_ = 0.830	*k* = −16→16
78666 measured reflections	*l* = −26→26

### Refinement

**Table:**

Refinement on *F*^2^	0 restraints
Least-squares matrix: full	Hydrogen site location: mixed
*R*[*F*^2^ > 2σ(*F*^2^)] = 0.053	H atoms treated by a mixture of independent and constrained refinement
*wR*(*F*^2^) = 0.135	*w* = 1/[σ^2^(*F*_o_^2^) + (0.0463*P*)^2^ + 1.0061*P*] where *P* = (*F*_o_^2^ + 2*F*_c_^2^)/3
*S* = 1.02	(Δ/σ)_max_ = 0.001
12002 reflections	Δρ_max_ = 0.32 e Å^−^^3^
535 parameters	Δρ_min_ = −0.35 e Å^−^^3^

### Special details

**Table:**

Geometry. All e.s.d.'s (except the e.s.d. in the dihedral angle between two l.s. planes) are estimated using the full covariance matrix. The cell e.s.d.'s are taken into account individually in the estimation of e.s.d.'s in distances, angles and torsion angles; correlations between e.s.d.'s in cell parameters are only used when they are defined by crystal symmetry. An approximate (isotropic) treatment of cell e.s.d.'s is used for estimating e.s.d.'s involving l.s. planes.

### Fractional atomic coordinates and isotropic or equivalent isotropic displacement parameters (Å^2^)

**Table:**

	*x*	*y*	*z*	*U*_iso_*/*U*_eq_	
S1A	0.08455 (7)	0.69136 (4)	0.16534 (3)	0.05868 (16)	
F1A	−0.31446 (17)	0.78739 (11)	0.47019 (10)	0.0905 (5)	
N1A	−0.10366 (15)	0.16736 (12)	0.33308 (8)	0.0362 (3)	
N2A	0.09099 (16)	0.39301 (12)	0.11449 (8)	0.0380 (3)	
N3A	0.12047 (15)	0.48279 (12)	0.07369 (8)	0.0365 (3)	
N4A	0.18107 (18)	0.64187 (14)	0.03380 (9)	0.0465 (4)	
H4AB	0.204 (2)	0.719 (2)	0.0296 (13)	0.073 (7)*	
N5A	0.20935 (18)	0.55200 (14)	−0.01880 (9)	0.0480 (4)	
C1A	−0.13898 (18)	0.14893 (14)	0.40148 (9)	0.0362 (4)	
C2A	−0.1351 (2)	0.05110 (16)	0.43631 (10)	0.0456 (4)	
H2AA	−0.1068	−0.0177	0.4130	0.055*	
C3A	−0.1746 (2)	0.05986 (18)	0.50619 (11)	0.0523 (5)	
H3AA	−0.1719	−0.0042	0.5304	0.063*	
C4A	−0.2188 (2)	0.16190 (18)	0.54209 (11)	0.0514 (5)	
H4AA	−0.2443	0.1647	0.5895	0.062*	
C5A	−0.22476 (19)	0.25805 (16)	0.50808 (10)	0.0437 (4)	
H5AA	−0.2552	0.3256	0.5317	0.052*	
C6A	−0.18407 (17)	0.25233 (14)	0.43690 (9)	0.0347 (3)	
C7A	−0.17501 (16)	0.33564 (14)	0.38866 (9)	0.0332 (3)	
C8A	−0.12480 (17)	0.28220 (14)	0.32566 (9)	0.0327 (3)	
C9A	−0.09289 (17)	0.34389 (14)	0.26649 (9)	0.0353 (3)	
H9AA	−0.1367	0.4096	0.2654	0.042*	
C10A	−0.01208 (18)	0.32505 (14)	0.21247 (9)	0.0370 (4)	
H10A	0.0286	0.2569	0.2059	0.044*	
C11A	0.01137 (18)	0.40985 (14)	0.16505 (9)	0.0362 (4)	
H11A	−0.0310	0.4771	0.1708	0.043*	
C12A	0.12757 (19)	0.60532 (15)	0.09206 (10)	0.0389 (4)	
C13A	0.17236 (19)	0.45754 (16)	0.00762 (10)	0.0423 (4)	
H13A	0.1800	0.3809	−0.0155	0.051*	
C14A	−0.07844 (19)	0.07247 (14)	0.27346 (10)	0.0394 (4)	
H14A	−0.0741	0.1071	0.2287	0.047*	
C15A	0.0590 (2)	0.0347 (2)	0.28984 (13)	0.0601 (6)	
H15A	0.0701	−0.0299	0.2517	0.090*	
H15B	0.0631	0.0092	0.3367	0.090*	
H15C	0.1309	0.1009	0.2916	0.090*	
C16A	−0.1958 (2)	−0.03206 (18)	0.25663 (12)	0.0545 (5)	
H16A	−0.1871	−0.0821	0.2106	0.082*	
H16B	−0.2813	−0.0028	0.2531	0.082*	
H16C	−0.1931	−0.0774	0.2956	0.082*	
C17A	−0.21013 (17)	0.45672 (14)	0.40646 (9)	0.0349 (3)	
C18A	−0.34346 (19)	0.47005 (16)	0.41822 (11)	0.0434 (4)	
H18A	−0.4101	0.4025	0.4124	0.052*	
C19A	−0.3798 (2)	0.58122 (18)	0.43839 (12)	0.0517 (5)	
H19A	−0.4699	0.5893	0.4453	0.062*	
C20A	−0.2803 (2)	0.67830 (17)	0.44782 (12)	0.0550 (5)	
C21A	−0.1487 (2)	0.67053 (18)	0.43688 (15)	0.0667 (6)	
H21A	−0.0829	0.7389	0.4434	0.080*	
C22A	−0.1138 (2)	0.55868 (17)	0.41574 (13)	0.0550 (5)	
H22A	−0.0238	0.5523	0.4077	0.066*	
S1B	0.68905 (7)	0.83235 (5)	0.03129 (3)	0.06422 (17)	
F1B	0.46558 (19)	0.78643 (12)	0.34049 (10)	0.0933 (5)	
N1B	0.49537 (16)	1.38803 (13)	0.20432 (8)	0.0401 (3)	
N2B	0.75225 (17)	1.11714 (12)	−0.00035 (8)	0.0452 (4)	
N3B	0.81061 (17)	1.01944 (12)	−0.03051 (8)	0.0432 (4)	
N4B	0.8607 (2)	0.85069 (15)	−0.07253 (10)	0.0563 (5)	
H4BB	0.866 (3)	0.773 (2)	−0.0831 (14)	0.080 (8)*	
N5B	0.9290 (2)	0.92793 (15)	−0.11091 (10)	0.0652 (5)	
C1B	0.40567 (18)	1.41730 (15)	0.25539 (9)	0.0383 (4)	
C2B	0.3447 (2)	1.51889 (16)	0.27086 (10)	0.0454 (4)	
H2BA	0.3608	1.5806	0.2447	0.054*	
C3B	0.2599 (2)	1.52490 (18)	0.32609 (11)	0.0500 (5)	
H3BA	0.2178	1.5917	0.3370	0.060*	
C4B	0.23559 (19)	1.43340 (18)	0.36616 (11)	0.0480 (5)	
H4BA	0.1802	1.4413	0.4042	0.058*	
C5B	0.29213 (18)	1.33217 (17)	0.35015 (10)	0.0424 (4)	
H5BA	0.2738	1.2706	0.3763	0.051*	
C6B	0.37813 (17)	1.32248 (15)	0.29381 (9)	0.0368 (4)	
C7B	0.45102 (17)	1.23172 (15)	0.26345 (9)	0.0365 (4)	
C8B	0.52169 (18)	1.27408 (15)	0.20931 (9)	0.0373 (4)	
C9B	0.60246 (18)	1.20425 (15)	0.16399 (10)	0.0391 (4)	
H9BA	0.6333	1.1443	0.1855	0.047*	
C10B	0.6420 (2)	1.20839 (15)	0.09709 (10)	0.0420 (4)	
H10B	0.6236	1.2692	0.0725	0.050*	
C11B	0.71322 (19)	1.11722 (15)	0.06399 (10)	0.0408 (4)	
H11B	0.7314	1.0574	0.0895	0.049*	
C12B	0.7857 (2)	0.90104 (15)	−0.02282 (10)	0.0437 (4)	
C13B	0.8952 (3)	1.02883 (18)	−0.08422 (12)	0.0575 (6)	
H13B	0.9251	1.0998	−0.0998	0.069*	
C14B	0.5777 (2)	1.47295 (16)	0.16779 (10)	0.0432 (4)	
H14B	0.6475	1.4314	0.1454	0.052*	
C15B	0.4908 (3)	1.5060 (2)	0.10514 (12)	0.0648 (6)	
H15D	0.5475	1.5581	0.0805	0.097*	
H15E	0.4198	1.5459	0.1248	0.097*	
H15F	0.4503	1.4350	0.0705	0.097*	
C16B	0.6536 (2)	1.58146 (18)	0.22234 (13)	0.0576 (5)	
H16D	0.7206	1.6251	0.1983	0.086*	
H16E	0.6987	1.5561	0.2636	0.086*	
H16F	0.5896	1.6318	0.2396	0.086*	
C17B	0.45015 (17)	1.11375 (15)	0.28393 (10)	0.0381 (4)	
C18B	0.4860 (2)	1.10356 (16)	0.35652 (11)	0.0457 (4)	
H18B	0.5065	1.1721	0.3929	0.055*	
C19B	0.4921 (2)	0.99369 (19)	0.37605 (13)	0.0559 (5)	
H19B	0.5177	0.9876	0.4247	0.067*	
C20B	0.4592 (2)	0.89457 (17)	0.32175 (15)	0.0586 (6)	
C21B	0.4200 (2)	0.89917 (18)	0.25037 (14)	0.0615 (6)	
H21B	0.3967	0.8296	0.2149	0.074*	
C22B	0.4154 (2)	1.00955 (17)	0.23120 (12)	0.0504 (5)	
H22B	0.3887	1.0140	0.1824	0.060*	

### Atomic displacement parameters (Å^2^)

**Table:**

	*U*^11^	*U*^22^	*U*^33^	*U*^12^	*U*^13^	*U*^23^
S1A	0.0947 (4)	0.0325 (2)	0.0520 (3)	0.0121 (2)	0.0265 (3)	0.0061 (2)
F1A	0.0981 (11)	0.0402 (7)	0.1313 (14)	0.0313 (7)	0.0106 (10)	−0.0051 (8)
N1A	0.0483 (8)	0.0295 (7)	0.0346 (7)	0.0111 (6)	0.0152 (6)	0.0072 (5)
N2A	0.0550 (9)	0.0260 (6)	0.0376 (7)	0.0088 (6)	0.0146 (7)	0.0121 (6)
N3A	0.0479 (8)	0.0286 (7)	0.0376 (7)	0.0096 (6)	0.0131 (6)	0.0114 (6)
N4A	0.0640 (10)	0.0333 (8)	0.0484 (9)	0.0096 (7)	0.0189 (8)	0.0175 (7)
N5A	0.0635 (10)	0.0417 (8)	0.0469 (9)	0.0156 (7)	0.0223 (8)	0.0166 (7)
C1A	0.0429 (9)	0.0326 (8)	0.0347 (8)	0.0064 (7)	0.0105 (7)	0.0074 (6)
C2A	0.0606 (12)	0.0351 (9)	0.0444 (10)	0.0101 (8)	0.0125 (9)	0.0112 (7)
C3A	0.0697 (13)	0.0430 (10)	0.0476 (11)	0.0036 (9)	0.0131 (10)	0.0193 (9)
C4A	0.0645 (13)	0.0532 (11)	0.0378 (10)	0.0021 (10)	0.0180 (9)	0.0124 (8)
C5A	0.0506 (11)	0.0411 (9)	0.0385 (9)	0.0034 (8)	0.0164 (8)	0.0028 (7)
C6A	0.0374 (8)	0.0314 (8)	0.0348 (8)	0.0031 (6)	0.0095 (7)	0.0042 (6)
C7A	0.0347 (8)	0.0297 (7)	0.0358 (8)	0.0052 (6)	0.0096 (7)	0.0048 (6)
C8A	0.0368 (8)	0.0282 (7)	0.0348 (8)	0.0066 (6)	0.0088 (7)	0.0071 (6)
C9A	0.0427 (9)	0.0286 (7)	0.0371 (9)	0.0088 (7)	0.0087 (7)	0.0082 (6)
C10A	0.0492 (10)	0.0281 (8)	0.0371 (9)	0.0092 (7)	0.0111 (7)	0.0094 (6)
C11A	0.0440 (9)	0.0293 (8)	0.0379 (9)	0.0084 (7)	0.0091 (7)	0.0092 (6)
C12A	0.0477 (10)	0.0298 (8)	0.0430 (9)	0.0088 (7)	0.0094 (8)	0.0127 (7)
C13A	0.0536 (11)	0.0370 (9)	0.0423 (10)	0.0131 (8)	0.0180 (8)	0.0126 (7)
C14A	0.0531 (10)	0.0301 (8)	0.0365 (9)	0.0092 (7)	0.0146 (8)	0.0038 (7)
C15A	0.0558 (12)	0.0563 (12)	0.0698 (14)	0.0210 (10)	0.0184 (11)	0.0006 (10)
C16A	0.0670 (13)	0.0414 (10)	0.0508 (11)	0.0011 (9)	0.0041 (10)	0.0046 (9)
C17A	0.0406 (9)	0.0300 (8)	0.0343 (8)	0.0070 (7)	0.0084 (7)	0.0041 (6)
C18A	0.0397 (9)	0.0354 (9)	0.0527 (11)	0.0045 (7)	0.0058 (8)	0.0024 (8)
C19A	0.0455 (11)	0.0490 (11)	0.0618 (13)	0.0183 (9)	0.0090 (9)	0.0032 (9)
C20A	0.0655 (13)	0.0330 (9)	0.0664 (13)	0.0190 (9)	0.0055 (11)	0.0010 (9)
C21A	0.0585 (13)	0.0295 (9)	0.107 (2)	0.0000 (9)	0.0110 (13)	0.0033 (11)
C22A	0.0391 (10)	0.0373 (10)	0.0883 (16)	0.0051 (8)	0.0153 (10)	0.0083 (10)
S1B	0.0919 (4)	0.0371 (3)	0.0697 (4)	0.0092 (3)	0.0418 (3)	0.0115 (2)
F1B	0.1249 (13)	0.0413 (7)	0.1258 (14)	0.0180 (8)	0.0379 (11)	0.0337 (8)
N1B	0.0509 (9)	0.0360 (7)	0.0381 (8)	0.0176 (6)	0.0147 (7)	0.0067 (6)
N2B	0.0683 (10)	0.0299 (7)	0.0451 (8)	0.0209 (7)	0.0244 (8)	0.0081 (6)
N3B	0.0665 (10)	0.0301 (7)	0.0410 (8)	0.0192 (7)	0.0242 (7)	0.0100 (6)
N4B	0.0911 (14)	0.0338 (8)	0.0544 (10)	0.0246 (9)	0.0361 (9)	0.0113 (7)
N5B	0.1056 (15)	0.0434 (9)	0.0627 (11)	0.0304 (10)	0.0506 (11)	0.0185 (8)
C1B	0.0421 (9)	0.0383 (9)	0.0343 (8)	0.0118 (7)	0.0076 (7)	0.0003 (7)
C2B	0.0550 (11)	0.0393 (9)	0.0434 (10)	0.0172 (8)	0.0089 (8)	0.0027 (8)
C3B	0.0521 (11)	0.0485 (11)	0.0484 (11)	0.0216 (9)	0.0076 (9)	−0.0057 (9)
C4B	0.0422 (10)	0.0556 (11)	0.0439 (10)	0.0119 (8)	0.0127 (8)	−0.0043 (9)
C5B	0.0403 (9)	0.0449 (10)	0.0403 (9)	0.0066 (8)	0.0089 (7)	0.0012 (8)
C6B	0.0365 (9)	0.0371 (8)	0.0348 (8)	0.0073 (7)	0.0051 (7)	−0.0004 (7)
C7B	0.0396 (9)	0.0344 (8)	0.0350 (8)	0.0086 (7)	0.0070 (7)	0.0017 (7)
C8B	0.0432 (9)	0.0342 (8)	0.0370 (9)	0.0133 (7)	0.0101 (7)	0.0049 (7)
C9B	0.0471 (10)	0.0335 (8)	0.0397 (9)	0.0144 (7)	0.0114 (8)	0.0050 (7)
C10B	0.0567 (11)	0.0332 (8)	0.0405 (9)	0.0178 (8)	0.0150 (8)	0.0059 (7)
C11B	0.0548 (11)	0.0313 (8)	0.0401 (9)	0.0142 (7)	0.0143 (8)	0.0073 (7)
C12B	0.0632 (12)	0.0309 (8)	0.0409 (9)	0.0150 (8)	0.0161 (8)	0.0062 (7)
C13B	0.0915 (16)	0.0388 (10)	0.0548 (12)	0.0238 (10)	0.0413 (11)	0.0166 (9)
C14B	0.0551 (11)	0.0391 (9)	0.0408 (9)	0.0166 (8)	0.0158 (8)	0.0096 (7)
C15B	0.0834 (16)	0.0719 (15)	0.0486 (12)	0.0267 (13)	0.0121 (11)	0.0237 (11)
C16B	0.0616 (13)	0.0447 (11)	0.0674 (14)	0.0100 (9)	0.0150 (11)	0.0081 (10)
C17B	0.0349 (8)	0.0329 (8)	0.0461 (10)	0.0047 (7)	0.0108 (7)	0.0037 (7)
C18B	0.0521 (11)	0.0359 (9)	0.0477 (10)	0.0006 (8)	0.0119 (8)	0.0062 (8)
C19B	0.0648 (13)	0.0498 (11)	0.0580 (12)	0.0071 (10)	0.0150 (10)	0.0215 (10)
C20B	0.0613 (13)	0.0345 (10)	0.0853 (17)	0.0063 (9)	0.0265 (12)	0.0185 (10)
C21B	0.0618 (13)	0.0336 (10)	0.0814 (17)	−0.0006 (9)	0.0130 (12)	−0.0057 (10)
C22B	0.0521 (11)	0.0423 (10)	0.0519 (11)	0.0050 (8)	0.0039 (9)	−0.0015 (8)

### Geometric parameters (Å, º)

**Table:**

S1A—C12A	1.6621 (18)	S1B—C12B	1.6618 (19)
F1A—C20A	1.356 (2)	F1B—C20B	1.359 (2)
N1A—C1A	1.382 (2)	N1B—C1B	1.386 (2)
N1A—C8A	1.399 (2)	N1B—C8B	1.394 (2)
N1A—C14A	1.479 (2)	N1B—C14B	1.473 (2)
N2A—C11A	1.283 (2)	N2B—C11B	1.281 (2)
N2A—N3A	1.3940 (18)	N2B—N3B	1.3955 (18)
N3A—C13A	1.368 (2)	N3B—C13B	1.362 (2)
N3A—C12A	1.385 (2)	N3B—C12B	1.386 (2)
N4A—C12A	1.344 (2)	N4B—C12B	1.341 (2)
N4A—N5A	1.372 (2)	N4B—N5B	1.371 (2)
N4A—H4AB	0.91 (3)	N4B—H4BB	0.90 (3)
N5A—C13A	1.287 (2)	N5B—C13B	1.287 (2)
C1A—C2A	1.395 (2)	C1B—C2B	1.394 (2)
C1A—C6A	1.411 (2)	C1B—C6B	1.409 (2)
C2A—C3A	1.373 (3)	C2B—C3B	1.377 (3)
C2A—H2AA	0.9300	C2B—H2BA	0.9300
C3A—C4A	1.396 (3)	C3B—C4B	1.394 (3)
C3A—H3AA	0.9300	C3B—H3BA	0.9300
C4A—C5A	1.371 (3)	C4B—C5B	1.367 (3)
C4A—H4AA	0.9300	C4B—H4BA	0.9300
C5A—C6A	1.402 (2)	C5B—C6B	1.402 (2)
C5A—H5AA	0.9300	C5B—H5BA	0.9300
C6A—C7A	1.417 (2)	C6B—C7B	1.423 (2)
C7A—C8A	1.388 (2)	C7B—C8B	1.385 (2)
C7A—C17A	1.478 (2)	C7B—C17B	1.473 (2)
C8A—C9A	1.436 (2)	C8B—C9B	1.437 (2)
C9A—C10A	1.342 (2)	C9B—C10B	1.335 (2)
C9A—H9AA	0.9300	C9B—H9BA	0.9300
C10A—C11A	1.429 (2)	C10B—C11B	1.432 (2)
C10A—H10A	0.9300	C10B—H10B	0.9300
C11A—H11A	0.9300	C11B—H11B	0.9300
C13A—H13A	0.9300	C13B—H13B	0.9300
C14A—C15A	1.514 (3)	C14B—C15B	1.518 (3)
C14A—C16A	1.518 (3)	C14B—C16B	1.520 (3)
C14A—H14A	0.9800	C14B—H14B	0.9800
C15A—H15A	0.9600	C15B—H15D	0.9600
C15A—H15B	0.9600	C15B—H15E	0.9600
C15A—H15C	0.9600	C15B—H15F	0.9600
C16A—H16A	0.9600	C16B—H16D	0.9600
C16A—H16B	0.9600	C16B—H16E	0.9600
C16A—H16C	0.9600	C16B—H16F	0.9600
C17A—C22A	1.379 (2)	C17B—C18B	1.386 (3)
C17A—C18A	1.386 (2)	C17B—C22B	1.389 (2)
C18A—C19A	1.381 (2)	C18B—C19B	1.384 (3)
C18A—H18A	0.9300	C18B—H18B	0.9300
C19A—C20A	1.356 (3)	C19B—C20B	1.364 (3)
C19A—H19A	0.9300	C19B—H19B	0.9300
C20A—C21A	1.353 (3)	C20B—C21B	1.354 (3)
C21A—C22A	1.385 (3)	C21B—C22B	1.385 (3)
C21A—H21A	0.9300	C21B—H21B	0.9300
C22A—H22A	0.9300	C22B—H22B	0.9300
			
C1A—N1A—C8A	107.86 (13)	C1B—N1B—C8B	107.49 (14)
C1A—N1A—C14A	124.51 (13)	C1B—N1B—C14B	125.46 (14)
C8A—N1A—C14A	126.36 (13)	C8B—N1B—C14B	124.92 (14)
C11A—N2A—N3A	117.96 (13)	C11B—N2B—N3B	116.57 (14)
C13A—N3A—C12A	108.07 (14)	C13B—N3B—C12B	108.25 (14)
C13A—N3A—N2A	119.78 (13)	C13B—N3B—N2B	120.17 (14)
C12A—N3A—N2A	131.35 (14)	C12B—N3B—N2B	131.03 (15)
C12A—N4A—N5A	114.68 (15)	C12B—N4B—N5B	114.71 (15)
C12A—N4A—H4AB	124.6 (16)	C12B—N4B—H4BB	125.5 (17)
N5A—N4A—H4AB	120.5 (16)	N5B—N4B—H4BB	119.8 (17)
C13A—N5A—N4A	103.21 (14)	C13B—N5B—N4B	103.27 (15)
N1A—C1A—C2A	131.02 (15)	N1B—C1B—C2B	130.38 (17)
N1A—C1A—C6A	108.27 (14)	N1B—C1B—C6B	108.73 (14)
C2A—C1A—C6A	120.71 (15)	C2B—C1B—C6B	120.88 (16)
C3A—C2A—C1A	117.77 (17)	C3B—C2B—C1B	117.91 (18)
C3A—C2A—H2AA	121.1	C3B—C2B—H2BA	121.0
C1A—C2A—H2AA	121.1	C1B—C2B—H2BA	121.0
C2A—C3A—C4A	122.14 (18)	C2B—C3B—C4B	121.70 (17)
C2A—C3A—H3AA	118.9	C2B—C3B—H3BA	119.1
C4A—C3A—H3AA	118.9	C4B—C3B—H3BA	119.1
C5A—C4A—C3A	120.66 (17)	C5B—C4B—C3B	120.80 (17)
C5A—C4A—H4AA	119.7	C5B—C4B—H4BA	119.6
C3A—C4A—H4AA	119.7	C3B—C4B—H4BA	119.6
C4A—C5A—C6A	118.62 (17)	C4B—C5B—C6B	119.06 (18)
C4A—C5A—H5AA	120.7	C4B—C5B—H5BA	120.5
C6A—C5A—H5AA	120.7	C6B—C5B—H5BA	120.5
C5A—C6A—C1A	120.09 (15)	C5B—C6B—C1B	119.59 (15)
C5A—C6A—C7A	132.27 (15)	C5B—C6B—C7B	133.36 (17)
C1A—C6A—C7A	107.63 (14)	C1B—C6B—C7B	107.05 (14)
C8A—C7A—C6A	106.91 (14)	C8B—C7B—C6B	107.00 (15)
C8A—C7A—C17A	128.82 (14)	C8B—C7B—C17B	125.95 (15)
C6A—C7A—C17A	124.25 (14)	C6B—C7B—C17B	127.04 (15)
C7A—C8A—N1A	109.33 (13)	C7B—C8B—N1B	109.70 (14)
C7A—C8A—C9A	122.57 (14)	C7B—C8B—C9B	122.96 (15)
N1A—C8A—C9A	127.96 (14)	N1B—C8B—C9B	127.25 (15)
C10A—C9A—C8A	132.44 (15)	C10B—C9B—C8B	132.70 (16)
C10A—C9A—H9AA	113.8	C10B—C9B—H9BA	113.7
C8A—C9A—H9AA	113.8	C8B—C9B—H9BA	113.7
C9A—C10A—C11A	119.92 (15)	C9B—C10B—C11B	118.64 (16)
C9A—C10A—H10A	120.0	C9B—C10B—H10B	120.7
C11A—C10A—H10A	120.0	C11B—C10B—H10B	120.7
N2A—C11A—C10A	119.84 (15)	N2B—C11B—C10B	120.80 (16)
N2A—C11A—H11A	120.1	N2B—C11B—H11B	119.6
C10A—C11A—H11A	120.1	C10B—C11B—H11B	119.6
N4A—C12A—N3A	101.82 (14)	N4B—C12B—N3B	101.69 (15)
N4A—C12A—S1A	126.62 (13)	N4B—C12B—S1B	126.76 (14)
N3A—C12A—S1A	131.56 (13)	N3B—C12B—S1B	131.55 (13)
N5A—C13A—N3A	112.20 (16)	N5B—C13B—N3B	112.06 (17)
N5A—C13A—H13A	123.9	N5B—C13B—H13B	124.0
N3A—C13A—H13A	123.9	N3B—C13B—H13B	124.0
N1A—C14A—C15A	111.43 (16)	N1B—C14B—C15B	110.91 (17)
N1A—C14A—C16A	111.89 (15)	N1B—C14B—C16B	112.39 (15)
C15A—C14A—C16A	112.67 (16)	C15B—C14B—C16B	112.68 (17)
N1A—C14A—H14A	106.8	N1B—C14B—H14B	106.8
C15A—C14A—H14A	106.8	C15B—C14B—H14B	106.8
C16A—C14A—H14A	106.8	C16B—C14B—H14B	106.8
C14A—C15A—H15A	109.5	C14B—C15B—H15D	109.5
C14A—C15A—H15B	109.5	C14B—C15B—H15E	109.5
H15A—C15A—H15B	109.5	H15D—C15B—H15E	109.5
C14A—C15A—H15C	109.5	C14B—C15B—H15F	109.5
H15A—C15A—H15C	109.5	H15D—C15B—H15F	109.5
H15B—C15A—H15C	109.5	H15E—C15B—H15F	109.5
C14A—C16A—H16A	109.5	C14B—C16B—H16D	109.5
C14A—C16A—H16B	109.5	C14B—C16B—H16E	109.5
H16A—C16A—H16B	109.5	H16D—C16B—H16E	109.5
C14A—C16A—H16C	109.5	C14B—C16B—H16F	109.5
H16A—C16A—H16C	109.5	H16D—C16B—H16F	109.5
H16B—C16A—H16C	109.5	H16E—C16B—H16F	109.5
C22A—C17A—C18A	117.71 (16)	C18B—C17B—C22B	117.97 (17)
C22A—C17A—C7A	122.67 (15)	C18B—C17B—C7B	120.80 (15)
C18A—C17A—C7A	119.54 (15)	C22B—C17B—C7B	121.23 (17)
C19A—C18A—C17A	121.63 (17)	C19B—C18B—C17B	121.48 (18)
C19A—C18A—H18A	119.2	C19B—C18B—H18B	119.3
C17A—C18A—H18A	119.2	C17B—C18B—H18B	119.3
C20A—C19A—C18A	118.09 (18)	C20B—C19B—C18B	118.0 (2)
C20A—C19A—H19A	121.0	C20B—C19B—H19B	121.0
C18A—C19A—H19A	121.0	C18B—C19B—H19B	121.0
C21A—C20A—C19A	122.76 (18)	C21B—C20B—F1B	118.8 (2)
C21A—C20A—F1A	118.94 (19)	C21B—C20B—C19B	122.98 (19)
C19A—C20A—F1A	118.30 (19)	F1B—C20B—C19B	118.3 (2)
C20A—C21A—C22A	118.65 (19)	C20B—C21B—C22B	118.66 (19)
C20A—C21A—H21A	120.7	C20B—C21B—H21B	120.7
C22A—C21A—H21A	120.7	C22B—C21B—H21B	120.7
C17A—C22A—C21A	121.14 (18)	C21B—C22B—C17B	120.9 (2)
C17A—C22A—H22A	119.4	C21B—C22B—H22B	119.6
C21A—C22A—H22A	119.4	C17B—C22B—H22B	119.6
			
C11A—N2A—N3A—C13A	−162.30 (17)	C11B—N2B—N3B—C13B	−157.5 (2)
C11A—N2A—N3A—C12A	29.3 (3)	C11B—N2B—N3B—C12B	32.0 (3)
C12A—N4A—N5A—C13A	0.0 (2)	C12B—N4B—N5B—C13B	0.0 (3)
C8A—N1A—C1A—C2A	−178.94 (19)	C8B—N1B—C1B—C2B	−177.88 (19)
C14A—N1A—C1A—C2A	13.3 (3)	C14B—N1B—C1B—C2B	18.1 (3)
C8A—N1A—C1A—C6A	0.66 (19)	C8B—N1B—C1B—C6B	1.4 (2)
C14A—N1A—C1A—C6A	−167.09 (15)	C14B—N1B—C1B—C6B	−162.60 (16)
N1A—C1A—C2A—C3A	178.64 (19)	N1B—C1B—C2B—C3B	−179.10 (19)
C6A—C1A—C2A—C3A	−0.9 (3)	C6B—C1B—C2B—C3B	1.7 (3)
C1A—C2A—C3A—C4A	0.6 (3)	C1B—C2B—C3B—C4B	0.5 (3)
C2A—C3A—C4A—C5A	0.3 (3)	C2B—C3B—C4B—C5B	−2.1 (3)
C3A—C4A—C5A—C6A	−0.8 (3)	C3B—C4B—C5B—C6B	1.5 (3)
C4A—C5A—C6A—C1A	0.4 (3)	C4B—C5B—C6B—C1B	0.6 (3)
C4A—C5A—C6A—C7A	−178.17 (19)	C4B—C5B—C6B—C7B	−179.50 (19)
N1A—C1A—C6A—C5A	−179.20 (16)	N1B—C1B—C6B—C5B	178.37 (16)
C2A—C1A—C6A—C5A	0.4 (3)	C2B—C1B—C6B—C5B	−2.3 (3)
N1A—C1A—C6A—C7A	−0.31 (19)	N1B—C1B—C6B—C7B	−1.53 (19)
C2A—C1A—C6A—C7A	179.35 (17)	C2B—C1B—C6B—C7B	177.81 (17)
C5A—C6A—C7A—C8A	178.54 (19)	C5B—C6B—C7B—C8B	−178.80 (19)
C1A—C6A—C7A—C8A	−0.17 (19)	C1B—C6B—C7B—C8B	1.08 (19)
C5A—C6A—C7A—C17A	0.2 (3)	C5B—C6B—C7B—C17B	2.4 (3)
C1A—C6A—C7A—C17A	−178.53 (15)	C1B—C6B—C7B—C17B	−177.68 (17)
C6A—C7A—C8A—N1A	0.59 (19)	C6B—C7B—C8B—N1B	−0.3 (2)
C17A—C7A—C8A—N1A	178.84 (16)	C17B—C7B—C8B—N1B	178.53 (16)
C6A—C7A—C8A—C9A	−175.47 (16)	C6B—C7B—C8B—C9B	−177.13 (16)
C17A—C7A—C8A—C9A	2.8 (3)	C17B—C7B—C8B—C9B	1.7 (3)
C1A—N1A—C8A—C7A	−0.78 (19)	C1B—N1B—C8B—C7B	−0.7 (2)
C14A—N1A—C8A—C7A	166.68 (16)	C14B—N1B—C8B—C7B	163.39 (16)
C1A—N1A—C8A—C9A	175.00 (17)	C1B—N1B—C8B—C9B	176.01 (17)
C14A—N1A—C8A—C9A	−17.5 (3)	C14B—N1B—C8B—C9B	−19.9 (3)
C7A—C8A—C9A—C10A	158.93 (19)	C7B—C8B—C9B—C10B	155.7 (2)
N1A—C8A—C9A—C10A	−16.4 (3)	N1B—C8B—C9B—C10B	−20.6 (3)
C8A—C9A—C10A—C11A	−173.41 (18)	C8B—C9B—C10B—C11B	−174.33 (19)
N3A—N2A—C11A—C10A	−174.54 (15)	N3B—N2B—C11B—C10B	−174.83 (17)
C9A—C10A—C11A—N2A	178.49 (17)	C9B—C10B—C11B—N2B	179.52 (19)
N5A—N4A—C12A—N3A	−0.8 (2)	N5B—N4B—C12B—N3B	−0.6 (2)
N5A—N4A—C12A—S1A	178.96 (15)	N5B—N4B—C12B—S1B	178.81 (17)
C13A—N3A—C12A—N4A	1.21 (19)	C13B—N3B—C12B—N4B	1.0 (2)
N2A—N3A—C12A—N4A	170.61 (17)	N2B—N3B—C12B—N4B	172.32 (19)
C13A—N3A—C12A—S1A	−178.51 (16)	C13B—N3B—C12B—S1B	−178.40 (19)
N2A—N3A—C12A—S1A	−9.1 (3)	N2B—N3B—C12B—S1B	−7.1 (3)
N4A—N5A—C13A—N3A	0.8 (2)	N4B—N5B—C13B—N3B	0.7 (3)
C12A—N3A—C13A—N5A	−1.4 (2)	C12B—N3B—C13B—N5B	−1.1 (3)
N2A—N3A—C13A—N5A	−172.20 (16)	N2B—N3B—C13B—N5B	−173.56 (19)
C1A—N1A—C14A—C15A	−75.0 (2)	C1B—N1B—C14B—C15B	−78.5 (2)
C8A—N1A—C14A—C15A	119.51 (19)	C8B—N1B—C14B—C15B	120.25 (19)
C1A—N1A—C14A—C16A	52.1 (2)	C1B—N1B—C14B—C16B	48.7 (2)
C8A—N1A—C14A—C16A	−113.34 (19)	C8B—N1B—C14B—C16B	−112.61 (19)
C8A—C7A—C17A—C22A	−64.4 (3)	C8B—C7B—C17B—C18B	125.1 (2)
C6A—C7A—C17A—C22A	113.6 (2)	C6B—C7B—C17B—C18B	−56.4 (3)
C8A—C7A—C17A—C18A	119.0 (2)	C8B—C7B—C17B—C22B	−54.0 (3)
C6A—C7A—C17A—C18A	−63.0 (2)	C6B—C7B—C17B—C22B	124.5 (2)
C22A—C17A—C18A—C19A	0.2 (3)	C22B—C17B—C18B—C19B	2.2 (3)
C7A—C17A—C18A—C19A	176.91 (17)	C7B—C17B—C18B—C19B	−176.87 (18)
C17A—C18A—C19A—C20A	−1.1 (3)	C17B—C18B—C19B—C20B	−1.1 (3)
C18A—C19A—C20A—C21A	1.4 (4)	C18B—C19B—C20B—C21B	−0.7 (3)
C18A—C19A—C20A—F1A	−177.68 (19)	C18B—C19B—C20B—F1B	179.67 (19)
C19A—C20A—C21A—C22A	−0.5 (4)	F1B—C20B—C21B—C22B	−179.12 (19)
F1A—C20A—C21A—C22A	178.5 (2)	C19B—C20B—C21B—C22B	1.2 (3)
C18A—C17A—C22A—C21A	0.7 (3)	C20B—C21B—C22B—C17B	0.0 (3)
C7A—C17A—C22A—C21A	−176.0 (2)	C18B—C17B—C22B—C21B	−1.6 (3)
C20A—C21A—C22A—C17A	−0.5 (4)	C7B—C17B—C22B—C21B	177.44 (18)

### Hydrogen-bond geometry (Å, º)

*Cg*1 is the centroid of ring N3*A*–N5*A*/C12*A*/C13*A*, *Cg*2 is the centroid of ring C17*B*–C22*B*, and *Cg*3 is the centroid of ring C1*A*–C6*A*.

**Table:**

*D*—H···*A*	*D*—H	H···*A*	*D*···*A*	*D*—H···*A*
N4*A*—H4*AB*···N2*B*^i^	0.90 (2)	2.05 (2)	2.944 (2)	170 (2)
N4*B*—H4*BB*···N2*A*^ii^	0.89 (2)	2.02 (2)	2.906 (2)	170 (3)
C15*B*—H15*E*···*Cg*1^iii^	0.96	2.91	3.521 (3)	123
C16*A*—H16*B*···*Cg*2^iv^	0.96	2.87	3.716 (2)	148
C21*A*—H21*A*···*Cg*3^v^	0.93	2.90	3.668 (2)	140

Symmetry codes: (i) −*x*+1, −*y*+2, −*z*; (ii) −*x*+1, −*y*+1, −*z*; (iii) *x*, *y*+1, *z*; (iv) *x*−1, *y*−1, *z*; (v) −*x*, −*y*+1, −*z*+1.
